# Maternal Stress and Ethnic Disparities in Pre-Eclampsia: The Significance of a Migrant Perspective

**DOI:** 10.3390/jcm15134882

**Published:** 2026-06-23

**Authors:** Bavo Hendriks, Lidvine Ngonseu Harpi, An Van Berendoncks, Hilmar Bijma, Anita Banerjee, Dominique Mannaerts

**Affiliations:** 1 Department of Obstetrics and Gynaecology, Antwerp University Hospital, Drie Eikenstraat 655, 2650 Edegem, Belgium; dominique.mannaerts@uza.be; 2 Department of Medical Genetics, Antwerp University Hospital, Drie Eikenstraat 655, 2650 Edegem, Belgium; lidvine.ngonseuharpi@uza.be; 3 Department of Cardiology, Antwerp University Hospital, Drie Eikenstraat 655, 2650 Edegem, Belgium; an.vanberendoncks@uza.be; 4 Department of Obstetrics and Gynaecology, Division Obstetrics and Foetal Medicine, Erasmus MC University Medical Centre Rotterdam, Doctor Molewaterplein 40, 3015 GD Rotterdam, The Netherlands; h.bijma@erasmusmc.nl; 5 Department of Care Ethics, University of Humanistic Studies, Kromme Nieuwegracht 29, 3512 HD Utrecht, The Netherlands; 6 UK Maternal Cardiology Society, 9 Fitzroy Square, London W1T 5HW, UK; anita.banerjee@gstt.nhs.uk; 7 Women’s Health Services, Guy’s and St Thomas’ Hospital Foundation Trust, Department of Women and Children’s Health, School of Life Course and Population Sciences, King’s College London, Westminster Bridge Road, London SE1 7EH, UK; 8 Research Group ASTARC, Antwerp Surgical Training, Anatomy and Research Centre, University of Antwerp, Universiteitsplein 1, 2610 Antwerp, Belgium

**Keywords:** pre-eclampsia, maternal stress, allostatic load, migration, ethnicity, epigenetics, ecosocial theory

## Abstract

Persisting ethnic disparities in pre-eclampsia (PE), cardiovascular disease (CVD), and maternal mortality call for a paradigm shift in how ethnicity is understood as a risk factor for PE. Starting from a migrant perspective, we argue that the transgenerational experience of maternal stress within shared, yet dynamic ecosocial contexts can be linked to core pathophysiological features of PE. A growing body of evidence suggests how a vicious cycle of chronic maternal stress, cardiovascular dysfunction, placental ER stress, and endothelial dysfunction may serve as a catalyst for the transmission of altered cardiovascular and neuro-endocrine stress reactivity patterns across generations, with a seemingly important role for foetal programming and epigenetics. As these alterations in stress reactivity patterns have in turn been associated with an increased risk of PE and CVD later in life, the resulting transgenerational chain reaction may ultimately allow for ethnic disparities in PE to be traced back to historic, stressful moments in the shared ecosocial contexts of ethnic minority women. Reconceptualising ethnicity as a proxy for the stratified and embodied experience of transgenerational maternal stress within its unique ecosocial contexts, rather than a stand-alone, non-modifiable risk factor, will therefore open new directions for future research, clinical care, and policy interventions aimed at advancing maternal health equity.

## 1. Introduction

There is a need for a paradigm shift in our understanding of the pathophysiology of pre-eclampsia (PE) and its associated risk factors. Ethnicity is one of those risk factors that is poorly understood, yet at the same time supported by an extensive body of epidemiological evidence [1]. The association appears to be strongest for early-onset (i.e., diagnosis before 34 weeks of gestation) and late preterm PE (i.e., diagnosis before 37 weeks of gestation) [1], classified according to the 2021 revised International Society for the Study of Hypertension in Pregnancy (ISSHP) classification, diagnosis, and management recommendations for international practice [2]. The International Federation for Gynaecology and Obstetrics (FIGO) therefore recommends including maternal ethnicity in screening and prevention practices, such as the widely used, first-trimester combined risk assessment that integrates specific maternal characteristics with measured values of mean arterial pressure, placental growth factor (PLGF), and uterine artery pulsatility index (UTPI) [3]. The Foetal Medicine Foundation (FMF) risk calculator, for example, integrates these variables into a patient-specific risk for developing preterm PE [3]. First-trimester initiation of aspirin treatment in a minimum dosage of 100 mg a day can then significantly reduce the risk of developing preterm PE in high-risk women (i.e., calculated risk of 1 in 100 or more) [3]. As the increased risk for developing PE in women of Afro-Caribbean and South Asian descent “remains significant even after adjusting for other confounding risk factors for PE”, maternal ethnicity groups that are recommended to be included in this first-trimester screening algorithm are “White”, “Afro-Caribbean”, “South Asian”, and “Mixed” [3].

Meanwhile, the most recent, annual “Mothers and Babies: Reducing Risk through Audits and Confidential Enquiries across the UK” (MBRRACE-UK) report recorded a respective 1.3- to 2.3-fold increased risk of maternal mortality among Asian and Black women compared to White women [4]. The ongoing and concerning rise in the overall UK maternal death rate, independent of COVID-19-specific deaths, and cardiovascular disease (CVD) accounting for one of the main, single causes of indirect maternal deaths, further urge for new perspectives on the persisting ethnic disparities in PE, CVD, and maternal health [4].

In this essay, we argue how a migrant perspective on maternal stress exposure and reactivity allows us to move beyond the mere association of ethnicity and PE. Migrant women find themselves on a unique yet dynamic intersection of individual, interpersonal, and contextual factors that shape their eventual pregnancy experience and outcome [5,6,7,8]. Their stories of displacement therefore not only call for a most timely, ecosocial approach [9] to maternal stress and ethnic disparities in women’s cardiovascular health but also illuminate more integrated perspectives on PE pathophysiology that may open novel entry points in prevention and care.

The terms women and maternal health used throughout this essay emphasise the differential pathogenesis, presentation, and management of CVD in women compared to men. We agree with the Lancet Regional Health—Europe commission on inequalities and disparities in cardiovascular health that a focus on women is crucial to address the gaps in research and clinical practice that have historically overlooked these differences [10]. A similar bias, however, exists for people assigned female at birth who do not identify as women or with maternity as such, yet whose experiences of stress, pregnancy, and potentially forced displacement remain as valid to be included in our argument.

## 2. Migration, Ethnicity, and Maternal Stress: An Ecosocial Approach

The World Health Organisation (WHO) recently acknowledged restrictive migration policies as key determinants of migrant health [5]. Restrictive migration policies have, for example, been associated with poor mental health and “migration-related stress” [6], exposing pregnant migrant women to an increased risk of perinatal mental health disorders [7,8]. Stressful or traumatic pre-migration and transit experiences, sexual- and gender-based violence, as well as isolation, restricted access to obstetric care, lack of support, language barriers, and status insecurity in the host country are among the most reported, often being cumulative stressors in this context [7].

The dynamic, social, and political contexts of forced displacement thus serve as a collective example of extremely stressful environments that (pregnant) migrant women are required to navigate. Contrasting and competing political interests, institutional racism, and the operational neglect of vulnerable migrant populations in Europe are argued to maintain a gendered and racialised reality of unequal access to the European mobility infrastructure, driving Sub-Saharan African migrant women onto particularly dangerous trails of shifting vulnerabilities [11,12]. On arrival, Sub-Saharan African migrants are consistently found to be at a higher risk of developing PE compared to other migrant groups [13].

Several authors warn for considering ethnicity as a stand-alone, non-modifiable biological category, rather than a socio-cultural and political construct with deep historical roots [14]. With more genetic variation within than amongst ethnic groups, ethnicity too often remains an alarmingly imprecise label in clinical research and practice, perpetuating its colonial conception as a genetic proxy for biological differences in disease distributions [14,15]. On the other hand, social determinants of health alone do not seem to fully explain persisting ethnic disparities in PE and maternal mortality either [15,16].

Reviewing twenty three frameworks that describe the causal pathways to maternal health and wellbeing as a multifactorial process, a recent Lancet Global Health Series paper argues how maternal morbidity and mortality are not just biomedical problems, but layered outcomes of a complex interplay of superdeterminants (e.g., climate, ecosystem, culture, political and economic system), social determinants (e.g., living and working conditions), individual characteristics and health system features (e.g., access to care) [17]. In their argument, the authors refer to Nancy Krieger’s ecosocial theory of disease distributions [9,17]. This theory illuminates how individual health disparities are shaped by the “political economy and ecology” of the shared context in which people live, conceptualising these disparities to reveal “embodied truths” that have historically been passed on across generations [9]. The deliberate choice of the term “ecosocial” in Krieger’s theory conveys “the fundamental interdependence of societal and ecological contexts” [9]. “Eco” literally refers to the evolving ecosystems or “ecologies” that shape and are shaped by the continuous, cross-species interactions with the living world around them [9]. “Social” in turn refers to the “sociality” in these within- and cross-species interactions [9]. In humans, this also includes “society”: the formal and informal rules that govern the social spaces and contexts in which people live, reproduce, and die, and where they generate ideas and behaviours that again shape the societies and ecosystems of which they are part [9].

Even though ethnicity can be held as a shared social construct, its embodied, transgenerational experience in our societies seems to have wide-ranging biological health effects. Ethnic disparities in PE and CVD would therefore more fittingly benefit from a more context-specific approach that centres the embodied, ecosocial experience of ethnicity and its transgenerational effects in pregnancy in the link between maternal stress, whether migration-related or not, and PE pathophysiology.

## 3. Chronic Maternal Stress and Pre-Eclampsia: Potential Pathophysiological Pathways

Two theories on PE pathophysiology currently prevail: the two-stage and the cardiogenic theory [18]. The two-stage theory focuses on the placenta to explain early-onset PE and associated foetal growth restriction (FGR) in particular [1,18]. The two stages described in this theory consist of abnormal placentation and reduced placental perfusion, followed by maternal endothelial dysfunction [1,18]. An impaired spiral artery remodelling process in the first trimester of pregnancy leads to a reduced placental perfusion rate later in pregnancy [18]. The resulting, relative placental hypoxia subsequently induces an increased maternal production of soluble, anti-angiogenic factors (i.e., first stage) [18]. Their combined, anti-angiogenic effect results in systemic endothelial dysfunction, reducing endothelial NO production and finally increasing vascular permeability and causing hypertension (i.e., second stage) [18].

The cardiogenic theory starts from the overlap between the common risk factors for PE and CVD and focuses on how pregnancy exacerbates pre-existing cardiovascular dysfunction [18]. Pre-existing heart disease and systemic endothelial dysfunction have both been associated with impaired maternal adaptation to the vascular and metabolic “stress test” of pregnancy [18,19]. The physiological hemodynamic changes in pregnancy require a compensatory response from the maternal heart and blood vessels to ensure a stable placental perfusion, resulting in a relative left ventricle eccentric hypertrophy [18,19]. As myocardial structure and function return to the pre-pregnancy state after delivery, these physiological adaptations will lead to an improved cardiovascular response in subsequent pregnancies [18,19]. When this cardiovascular “training and recovery process” is impaired, primiparous women diagnosed with PE may still exhibit persistent left ventricle dysfunction in the post-partum period and thus may risk failing the cardiovascular stress test again in a subsequent pregnancy and/or developing CVD later in life [18,19].

### 3.1. Chronic Maternal Stress from Shared Ecosocial Experiences as a Potential Pathway for PE Risk Disparities

Bruce McEwen conceptualises stress as a “threat, real or implied, to the physiological or psychological integrity of an individual” that results in physiological, psychological and/or behavioural responses primarily aimed at maintaining homeostasis [20,21]. “Allostasis” is then defined as an adaptive, multisystem process that adjusts the internal environment to new “set points” of physiological parameters to prioritise survival and maintain “homeostasis through change” in the face of a stressful event or environment [20,21,22]. “Allostatic load” (AL) in turn refers to the cumulative, physiological “wear and tear” of chronically disturbed homeostasis, either through repeated or chronic stress exposure, and/or the lack of adaptation or the prolonged or inadequate response to stress [20,21]. As the physiological stress response involves a complex interaction between the neuro-endocrine, cardiovascular, and immune system, repeated, sustained, or chronic stress can therefore have harmful, biological effects on the body [21,22]. AL therefore offers a promising conceptual link between the multisystem, physiological impact of people’s dynamic, ecosocial contexts and maternal health outcomes like PE [21,22].

According to its original conceptual framework, the degree to which cumulative stress responses may result in AL accumulation thus depends on three main characteristics: their magnitude and frequency, the ability to deactivate the response once the stressor has ceased, and whether they are sufficient to cope with the stressor [21]. The classic distinction between “acute” versus “chronic” stress therefore becomes increasingly arbitrary, as both the emotional and physiological effects of an acute stressor can linger on and become chronic [21,23]. The recurrence of acute stress over time or when daily stress exposure is caused by the same ongoing situation could thus lead to increased AL and induce cumulative, and even transgenerational health effects [21,22].

Several measurable biomarkers or mediators of AL have furthermore been described: cortisol, dehydroepiandrosterone (DHEA), epinephrine, norepinephrine, total cholesterol (TC), glycosylated haemoglobin (HbA1c), resting systolic and diastolic blood pressure (SBP, DBP), body mass index (BMI), and waist–hip ratio (WHR) [21]. Quantifying the exact AL in an individual however remains challenging given its complex, physiological basis in a multisystem interaction, embedded within dynamic, ecosocial contexts, and in case of pregnancy, its trimester- or even month-specific physiological changes [21,22,24].

As conceptual qualities turn into new challenges, recent findings did reveal a significant association of individual AL components measured during the first trimester with hypertensive disorders in pregnancy (HDP) and higher overall AL levels in the second trimester with PE specifically [21]. Secondary analyses from a large, prospective, U.S.-based cohort study of pregnancy outcomes in nulliparous women evaluating the association between ethnicity, HDP, and subsequent CVD risk even revealed a significant average causal mediation effect of AL in observed HDP disparities between self-identified non-Hispanic Black and non-Hispanic White participants [25,26]. Consistently, regression-based AL levels were also significantly elevated in non-Hispanic Black compared to non-Hispanic White participants [25].

Even though a growing body of evidence does suggest a mediating role for AL between the “double stress test” of chronic maternal stress on top of the vascular and metabolic stress test of pregnancy on the one hand, and ethnic disparities in PE on the other, the exact, underlying physiological mechanisms in this relationship remain unclear. Furthermore, for these disparities to arise from a shared, and thus stratified ecosocial experience like ethnicity, the transgenerational inheritance of physiological stress reactivity patterns and AL accumulation also warrants further study.

### 3.2. Neuro-Endocrine Stress Reactivity

Neuro-endocrine stress reactivity generally becomes more attenuated in late pregnancy due to a suggested, physiological downregulation of the hypothalamic–pituitary–adrenal (HPA) axis or response system [27]. Core mechanisms in this downregulation are an enhanced, central neurosteroidogenesis, upregulated endogenous opioid expression, and adaptations in the higher brain regions, hypothalamus, and anterior pituitary [27]. One of the most well studied neurosteroids that potentiates the central gamma-aminobutyric acid (GABA)-mediated inhibition of stress-induced HPA axis activity is allopregnanolone, a metabolite of progesterone [27]. Acute physical or psychological stressors trigger an increase in both the circulating and brain levels of allopregnanolone [27]. Together with other central neurosteroids, allopregnanolone acts as a positive allosteric modulator of the GABA inhibitory neurons that target the corticotropin-releasing hormone (CRH) neurons in the medial parvocellular paraventricular nucleus (mpPVN) of the brain [27]. As progesterone levels rise during pregnancy, animal studies show how allopregnanolone brain levels peak and HPA axis responses to acute stressors become markedly suppressed towards the end of pregnancy [27].

This physiological “set point” of neurosteroid HPA axis activity modulation may be altered under chronic, psychological stress exposure [22,27]. Available data collected in prenatally stressed rodents not only indicate a reduced, central capacity for neurosteroidogenesis and HPA axis regulation in prenatally stressed rodents, but also an impaired glucocorticoid feedback control of the HPA axis and adrenocorticotropic hormone (ACTH) and corticosterone stress responses of greater amplitude and/or increased duration [27]. Foetal programming through prenatal glucocorticoid exposure and epigenetics are believed to alter this prenatal HPA axis “set point” or neuro-endocrine stress sensitivity already in utero with their effects on central neurosteroidogenesis, HPA function, and behaviour persisting into adulthood, although exact, underlying mechanisms again remain to be elucidated [27,28].

### 3.3. Cardiovascular Stress Reactivity

A growing body of evidence further supports an association between neuro-endocrine stress sensitivity and HDP [27,29]. As the HPA axis works in concert with the sympathetic nervous system in response to an acute stressor, causing an increase in both heart rate and blood pressure, chronic or repeated stress exposure has been hypothesised to cause further dysregulation of autonomic, cardiovascular stress responses that seemingly manifest as either “hyperreactivity” or “blunted reactivity” [23]. Either way, both reactivity patterns may further suggest a maladaptive response to the double stress test of chronic maternal stress, like migration-related stress in pregnancy.

With hyperreactivity patterns currently receiving the most attention in the cardiovascular health literature, some authors suggest distinguishing between either myocardial or vascular responders to external stressors [29]. The frequent and extended, autonomic peripherical vasoconstriction in the face of chronic stress have been associated with an increased resting systemic vascular resistance and endothelial dysfunction over time, two recurring features in both the two-stage and cardiogenic theories of PE pathogenesis [29]. A greater tendency towards vascular stress responding has furthermore been reported in non-Hispanic Black versus non-Hispanic White, non-pregnant individuals [30]. AL has consistently been observed to be a partial mediator between “race” or ethnicity and CVD risk, chronic hypertension, and metabolic disorder developing within two to seven years post-partum [26], features that may in turn increase the risk of failing the cardiovascular stress test again in a subsequent pregnancy and/or developing CVD later in life [18,19]. Research on the exact, underlying, and plausibly multisystem physiological mechanisms that link altered autonomic stress responses to HDP, subsequent CVD, and their transgenerational inheritance patterns, however, so far remains limited.

### 3.4. Transcriptomic Signatures of Maternal Stress and PE

Daughters or sisters of women with PE have been observed to be “three to four times more likely to develop the condition than women without a family history” [3]. Familial susceptibility to PE thus requires special consideration in our further understanding of ethnic disparities in PE. The mode of inheritance of PE risk and protective factors, however, seems to be as much a complex patchwork of different, interactive components as the multisystem origin of their mothers’ disease [3]. Genetic predisposition [31,32], or, for that matter, inherited yet dynamic ecosocial contexts and their net impact on the offspring’s AL accumulation, remain essential in delineating their actual risk of developing PE or CVD later in life. Meanwhile, neuro-endocrine and cardiovascular stress reactivity patterns in the offspring may be altered in utero through foetal programming, with effects that seem to persist into adulthood [22,27]. Emerging evidence further suggests that epigenetics may also play an important role in PE risk transmission, a process that is directly shaped by transgenerational psychosocial stress exposure within dynamic, ecosocial contexts [33].

Specific cell-free DNA methylation (cfDNAme) differences that were observed between control and PE pregnancies have, for example, led to the early validation of a novel first-trimester risk prediction model for early-onset PE [34]. By integrating cfDNAme analysis only with maternal characteristics, the combined risk score correctly identified 72% of patients with early-onset PE at 80% specificity, increasing screening sensitivity compared to risk estimation based on maternal characteristics alone by 29% [34]. To evaluate whether cfDNAme patterns also allow for risk stratification between heterogenous subgroups, the authors called for further evaluation in larger and ethnically more diverse cohorts, as their cohort mainly consisted of women of European descent [34].

A transcriptome-wide association study did identify specific, stress-induced placental transcriptomic signatures, with maternal stress operationalised this time as childhood traumatic events (CTE) and maternal prenatal stressful life events (MPSLE) [35]. A higher number of MPSLE appeared to be associated with specific, differentially expressed genes that play a critical role in placental modelling and core placental functions [35]. The combination of CTE and MPSLE could further be associated with altered placental gene expressions of several gene sets related to protein homeostasis, resulting in placental endoplasmic reticulum (ER) stress, whereas CTE alone were not associated with the differential expression of any single genes [35]. Placental ER stress has, in turn, been described to primarily impair placental development in vitro, disrupt the spiral artery remodelling process, and contribute to endothelial dysfunction, all important features of the two-stage theory on PE pathophysiology [18,35]. Endothelial dysfunction also recurs in the cardiogenic theory as an underlying mechanism for increased systemic vascular resistance and reduced vascular reactivity, impairing the cardiovascular stress response [18,19,36]. Through its intrinsic link with the oxidative stress pathway, ER stress can occur secondary to reduced placental perfusion, again a central feature in both the two-stage and cardiogenic theory, suggesting a vicious cycle of maternal stress, placental ER stress, and an impaired cardiovascular stress response [18,36]. As the reoccurrence of placental transcriptomic signatures of maternal stress may also vary across generations, their specific transgenerational inheritance patterns additionally require further investigation.

Finally, MicroRNAs (miRNAs) seem to play a central role in the epigenetic programming of the individual risk of developing PE and CVD later in life, through the post-transcriptional regulation of gene expression involved in placental development and vascular adaptation to pregnancy [37,38]. Circulating MiR-16 and MiR-200c have, for example, been observed to be down- and upregulated, respectively, in women with early-onset PE, correlating with impaired angiogenesis, endothelial dysfunction, and arterial stiffness [38].

The integration of maternal stress in future research on the role of pre- and post-translational epigenetic modifications in DNA methylation and MiRNA programming, respectively, in PE pathogenesis, clearly holds promising implications for our future understanding of the pathophysiological link between the transgenerational experience of maternal stress, (early-onset) PE, and CVD. Questions, however, remain on which exact modifications are stress-induced, how much they contribute to an individual’s eventual PE risk, whether they are inherited or driven by an inherited ecosocial environment, and how well they resonate with observed ethnic disparities in PE.

**Figure 1:**
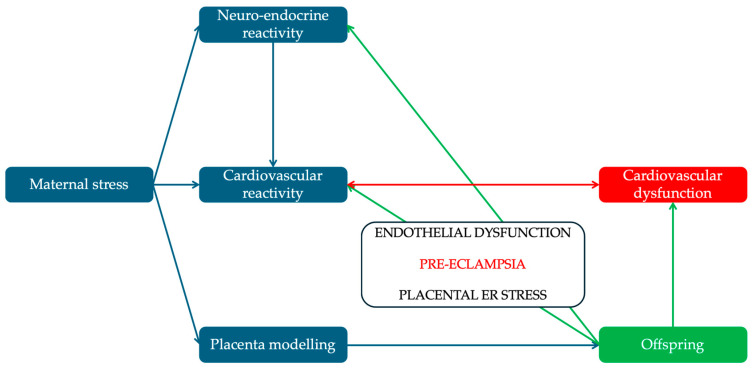
Maternal stress serving as a catalyst for the transgenerational transmission of altered stress reactivity and placental modelling patterns. As chronic maternal stress can increase the neuro-endocrine sensitivity to cumulative stressors, secondarily altered autonomic, cardiovascular stress reactivity patterns may increase the subsequent risk of a maladaptive response to the “double stress test” of chronic maternal stress and hemodynamic changes in pregnancy [23,27,29]. Cardiovascular hyperreactivity has been associated with increased systemic vascular resistance [23], whereas a history of CTE and MPSLE further seems to influence the transcription of critical genes involved in placental modelling and core placental functions [35]. These cardiovascular and placental effects have in turn been linked to increased placental ER stress and endothelial dysfunction, two recurrent, core elements in both the cardiogenic and two-stage theories on PE pathophysiology [18,23,35]. The cardiogenic theory further allows for a life course perspective where an impaired cardiovascular “training and recovery process” may potentiate another cycle of cardiovascular maladaptation and PE in a subsequent pregnancy (i.e., red, bidirectional arrow), especially when chronic stress conditions persist, increasing the risk of developing CVD later in life with every cycle [18,19]. Finally, neuro-endocrine and cardiovascular stress reactivity patterns in the offspring may be altered in utero through foetal programming, with effects that seem to persist into adulthood, potentially adding onto the female offspring’s risk of developing PE through a similar cascade of events (i.e., green arrows) [22,27,28].

Figure 1 summarises how the argued vicious cycle of maternal stress, cardiovascular dysfunction, placental ER stress, and endothelial dysfunction may serve as a catalyst for the transmission of altered neuro-endocrine stress and cardiovascular reactivity and placental modelling patterns across generations, with a seemingly important role for foetal programming in configuring the offspring’s baseline allostatic set point. This set point may then in turn contribute to their sensitivity for AL accumulation in their inherited, yet again dynamic, ecosocial environment and the cycle may repeat itself. The degree to which this cycle may be reiterated will of course always depend on a wide range of multilevel, life course factors—from ever-changing ecosocial environments and exposures to local health system features; from psychological coping mechanisms to internal physiology and epigenetics.

## 4. Discussion

Maternal morbidity and mortality represent layered, multisystem outcomes of a complex, intersectional interplay of both risk and protective factors [17]. The transgenerational health effects of chronic maternal stress and AL accumulation, however, seem to have been structurally overlooked in our understanding of ethnic disparities in PE. A growing body of evidence supports the hypothesis that chronic maternal stress may have contributed to vicious, transgenerational cycles, wherein PE incidence rates started to vary among groups that have historically shared and interacted with certain, stressful ecosocial environments [9,18,19,22,23,27,28,29,35]. Ethnic disparities in PE could therefore be considered as one of many embodied, ecosocial experiences ethnic minority women may share, each from their unique ecological, historical, political, social, and cultural network of “truths” [9].

Forced displacement furthermore entails a unique, dynamic ecosocial experience of rapidly changing social and political contexts that pregnant migrant women are also required to navigate. As current EU border and asylum policies shape a stratified experience of migration-related stress based on background and ethnicity [11,12], adherence to a migrant perspective allows for a most timely take on maternal stress, PE pathophysiology, and persisting ethnic disparities in women’s cardiovascular health.

Sub-Saharan African immigrant women in New York City have, for example, been found to be at a higher risk of developing PE than non-Hispanic, white American women, yet lower than in African American women [39]. Again, keeping the proposed, vicious pathophysiological cycle of maternal stress and PE in mind, an ecosocial approach may explain these maybe unexpected findings by suggesting that the historic experience of the United States ecosocial environment has predisposed African American women more to transgenerational AL accumulation and developing PE than the risk Sub-Saharan African immigrant women already face from their often challenging ecosocial backgrounds [9,14]. With a notorious history of slavery, the Jim Crow legislation, institutional racism, and ongoing discrimination, it can indeed be argued that African American women carry a historic, cumulative allostatic burden of altered neuro-endocrine and cardiovascular stress reactivity, and epigenetic changes in placental modelling patterns [9]. Meanwhile, a recent systematic review of natural experiments among migrant populations demonstrating detrimental health effects of migration and adverse post-migratory contexts largely refutes the often suggested “healthy migrant effect” [40] and raises further questions on whether, for example, colonisation, violence and conflict, and the institutional racism in both current migration policies as well as in our own health systems may add up to a similar transgenerational, allostatic burden among Sub-Saharan African migrants and their offspring in Europe [11,12,41].

While much of the evidence used to build our argument relies on associational research, animal models, and theoretical frameworks, it still reveals meaningful patterns that warrant further investigation and encourage the integration of epidemiology, structural determinants of health, and pathophysiology. While this essay is narrative in nature, a risk of selection and interpretation bias however requires full acknowledgement in the absence of a systematic literature review. We further decided to not include immune or metabolic factors in our argument. Although both systems constitute two key components of the multisystem, physiological stress response, research gaps on their moderating role in the stratified effect of maternal stress and PE currently hamper a more integrated analysis of the relationship between AL and ethnic disparities in HDP and women’s cardiovascular health.

Finally, an ecosocial approach to the shared, embodied experience of transgenerational stress may deepen our understanding of both ethnic disparities in HDP and PE pathophysiology but also risks further stigmatisation of ethnic minority women by trading the predominant biological or genetic determinism behind “ethnicity” in research and clinical practice for a new “stress determinism”. As (inherited) ecosocial contexts and their interaction with internal physiology are inherently dynamic, acknowledging both risk and potential resilience and community-based protective factors remain essential in respecting their reciprocal, net impact on the transgenerational risk of PE and CVD.

## 5. Conclusions and Future Directions

This essay conceptualises ethnicity more as a proxy for a shared, embodied experience of altered neuro-endocrine and cardiovascular stress reactivity patterns that can be passed on across generations, rather than a stand-alone risk factor that needs to be computed in first-trimester risk calculators. A growing body of evidence encourages a more ecosocial approach to observed ethnic disparities in PE risk, with available data ranging from a macrosystems level to the microlevel of gene expression and epigenetics. Adherence to a migrant perspective offers a timely case study that adds temporal depth to the developing notion of how shared, stressful ecosocial exposures before and during pregnancy may produce stratified, physiological differences in AL accumulation and stress reactivity—differences that may ultimately underpin and exacerbate existing ethnic disparities in PE and women’s cardiovascular health.

More robust, real-world, and prospective research is urgently needed on the multisystem links between stress exposure and physiological reactivity, before, during, and after pregnancy, and patients’ associated risk of developing PE and CVD later in life. Epigenetic links between maternal stress, cardiovascular and neuro-endocrine stress reactivity, placental modelling and PE, as well as their exact inheritance patterns also require further investigation. The current lack of consensus on how to define, screen for, measure and compare individual maternal stress exposures, past and present, AL, and stress reactivity profiles, however, continues to pose a primary challenge in generating coherent evidence to support person-centred PE screening, prevention, and management.

In summary, a more ecosocial approach to ethnic disparities in PE and women’s cardiovascular health may offer a novel and timely lens on maternal ethnicity as a risk factor with meaningful implications for future research, clinical care, and policy interventions. Integrating antenatal mental health screening and even epigenetic profiling into the first-trimester PE risk screening tool, as well as cardiovascular risk and AL profiling, could, for example, be concrete clinical steps forward in developing more person-centred screening and prevention strategies. Although health sectors do play a crucial role in reducing maternal health disparities, the consistent integration of both a “health in all policies” [42] and anti-discrimination approach in the design and implementation of social and migration policies may offer even greater potential for advancing maternal health equity.

For now, the main argument in this essay remains not to discard ethnicity as a risk factor for PE but to co-integrate maternal stress and AL in both research and maternity care. It is mainly a call for researchers and clinicians to engage in meaningful conversations on maternal stress, cardiovascular health, and family histories of PE with all their patients. A shift towards screening for and reducing maternal stress, rather than focusing on ethnicity as a stand-alone, non-modifiable risk factor, offers an important opportunity to work towards more community-based, integrated health services that provide accessible, person-centred, and respectful maternity care. Meanwhile, clinicians should continue to advocate for maternity services that are and feel safe for ethnic minority and migrant women, address institutional racism within their own health networks, push for diversity and representation in their workforce, and operationalise the fundamental right to universal access to healthcare.

## Data Availability

No new data were created or analysed in this study.

## Abbreviations

The following abbreviations are used in this manuscript:

ACTH	Adrenocorticotropic hormone
AL	Allostatic load
BMI	Body mass index
cfDNAme	Cell-free DNA methylation
CRH	Corticotropin releasing hormone
CTE	Childhood traumatic event
CVD	Cardiovascular disease
DBP	Diastolic blood pressure
DHEA	Dehydroepiandrosterone
ER	Endoplasmic reticulum
FGR	Foetal growth restriction
FIGO	International Federation for Gynaecology and Obstetrics
GABA	Gamma-aminobutyric acid
HbA1c	Glycosylated haemoglobin
HDP	Hypertensive disorders in pregnancy
HIC	Higher income countries
HPA	Hypothalamic–pituitary–adrenal
ISSHP	International Society for the Study of Hypertension in Pregnancy
LMIC	Lower- and middle-income countries
MBRRACE-UK	Mothers and Babies: Reducing Risk through Audits and Confidential Enquiries across the UK
miRNAs	MicroRNAs
mpPVN	Medial parvocellular paraventricular nucleus
MPSLE	Maternal prenatal stressful life events
NO	Nitric Oxide
PE	Pre-eclampsia
SBP	Systolic blood pressure
TC	Total cholesterol
WHO	World Health Organisation
WHR	Waist–hip ratio
